# 100 Disruptive Publications in Breast Cancer Research

**DOI:** 10.31557/APJCP.2021.22.8.2385

**Published:** 2021-08

**Authors:** Miles W Grunvald, Michael D Williams, Ruta D Rao, Cristina M O’Donoghue, Adan Z Becerra

**Affiliations:** 1 *Department of Surgery, Rush University Medical Center, Chicago, IL, 60654, USA. *; 2 *Department of Hematology and Oncology, Rush University Medical Center, Chicago, IL, USA. *

**Keywords:** Breast cancer, disruption, bibliometrics

## Abstract

**Background:**

Breast cancer has a rich history of research over the past 75 years. Many studies have had disruptive influences on the field itself. Our study employs a new, validated measurement to determine the most disruptive publications within the field of breast cancer.

**Materials and Methods:**

PubMed^®^ database was queried for articles between 1954-2014 related to breast cancer with in 21 different journals deemed important to the field. Articles were then scored for disruption and citation count. The top 100 most disruptive and cited publications were compiled and analyzed.

**Results:**

Disruption score was a distinct measurement from citation count and had low level of correlation. Disruptive publications tended to skew older with the median year of publication in 1977. The score identified a variety of study designs and publication types within multiple journals.

**Conclusions:**

Measurement of the disruptive quality of a publication is a new way to describe academic impact of a publication and is distinct from citation count. Used in conjunction with citation count in may give a more descriptive bibliometric assessment of the literature. Further exploration within the field of oncology is warranted.

## Introduction

Bibliometrics are used to analyze academic productivity and the impact of scientific publications(Borgman and Furner, 2005). The number of times an individual paper has been cited, known as the “citation count” has been the predominant metric for assessing the importance or utility of a publication to its field of study. However, this method has come under scrutiny for not fully describing the importance of some contributions to the areas of scientific exploration. Specifically, citation count assumes that all citations are equal and that they are all positive, demonstrating the quality/impactfullness of a publication. There is no qualifier in the citation count to explain why an individual study has received citations. Did the research introduce a groundbreaking technique that warranted its citation count? Or was the study a culmination of years of supporting research into a particular area? These questions are unanswered using citation count alone; new tools have been created to give a richer explanation of the impact of individual publications (Davis, 2008; Hendrix, 2008; Petersen et al., 2010; Pinski and Narin, 1976; Wu et al., 2019).

Measurements of the disruptive quality of a scientific publication are an emerging way to assess and capture academic achievement. Ideally scores of disruption capture the characteristic of “introducing something new that eclipses attention to previous work upon which it has built.” From this approach a disruptive paper could be described as one that is cited more frequently than its own references and, indeed, a disruptiveness scoring tool has been built based on this very concept.(Wu et al., 2019) Already disruption is being utilized to evaluate the body of literature in multiple surgical specialties including; general surgery, urology, and colorectal surgery(Becerra et al., 2020; Hansdorfer et al., 2021; Khusid et al., 2020).

Breast cancer research provides a rich substrate upon which this disruptiveness tool can be utilized. Recent history of breast cancer research and care has been marked by many clinical trials, changes in technology, and regular adoption of new standards of care (Ades et al., 2017; Mascaro et al., 2010). These characteristics serve as an interesting environment for the assessment of prior publications with new bibliometrics. We aim to benchmark the disruption score portfolio of breast surgery literature using a validated dataset of all PubMed® publications between 1954-2014. Correlation between publications captured using the disruption score to those captured using citation score will be conducted with the hypothesis that there will be a weak correlation and that the scores will be assessing different qualities of publications. 

## Materials and Methods

The PubMed^®^ database was queried for any articles containing the phrase “breast,” or “mammography,” in the title. These terms were chosen to be sensitive for articles related to breast cancer research with an understanding that it may lack some specificity. The search was further filtered by journals deemed most important to breast cancer care and research. These journals were selected by a multidisciplinary team of attending physicians that include a medical oncologist and surgical oncologist (supplement Table 1). After compiling the publication list from PubMed^®^, the disruption scores were obtained having been calculated using the method described by Wu (2019).

The disruption score is reported as a ratio and varies between -1 and +1. The score can be calculated using equation 1. Positive scores correspond to publications that disrupt science with those closer to +1 being the most disruptive. The papers were also analyzed for raw citation count. Publications were ranked by their disruption score and separately by citation count. The top 100 were selected for each category. These top 100 lists were interrogated, and information was obtained regarding study design and year of publication. The correlation coefficient between disruption score and citation count of the top 100 ranking papers in each category was calculated. Analysis was completed using Microsoft Excel and R Statistical software. A kernel density plot of the entire PubMed^®^ universe 1954-2014 was generated to visualize the distribution of disruption scores for all PubMed^®^ paper. The years 1954-2014 were chosen because these were the years that had calculated disruption scores in the publicly available dataset that we used. 

$\mathrm{Disruption}=\frac{\left(\left(\mathrm{\# of papers that cite the focal paper without citing any of its references}\right)-\left(\mathrm{\# of papers that cite the focal paper and cite any of its references}\right)\right)}{\left(\left(\mathrm{\# of citations of focal paper}\right)+\left(\mathrm{\# of papers that cite references from the paper without citing the focal paper}\right)\right)}$

Equation 1

**Figure 1:**
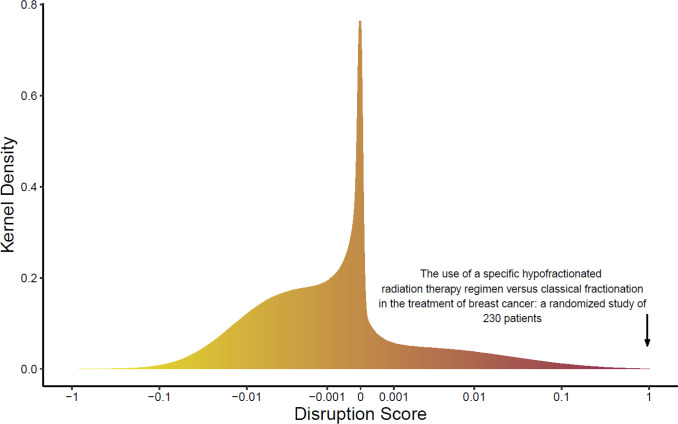
Kernel Density Plot of Disruptions Scores in the PubMed Universe 1954-2014

**Figure 2:**
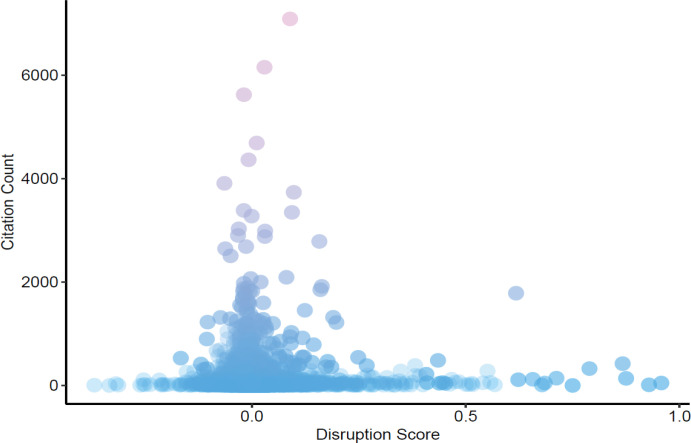
Distribution of Studies Based on Disruption Score and Citation Count

Disruption score=(((# of papers that cite the focal paper without citing any of its references) - (# of papers that cite the focal paper and cite any of its references)))/(((# of citations of focal paper) + (#of papers that cite references from the focal paper without citing the focal paper)) )

## Results

The PubMed^®^ search, using the criteria specified above, revealed 25,612 articles. The 100 most disruptive publications and 100 most cited papers in breast cancer care as determined in this study are listed in Table 1 and supplementary Table 2. A kernel density plot of disruption scores in the PubMed universe is shown in Figure 1. The average breast cancer paper had a disruption score of -0.00115 while the entire PubMed universe had an average of -0.00055; the top 100 most disruptive breast cancer papers were more disruptive than 99.9% of the Pubmed universe. 

15 of the 21 journals had articles with the 100 most disruptive list; the most represented journal was “Cancer” followed by the “Lancet” (supplementary Table 3). Disruptive publications ranged from having 1 to 1,785 citations. The most cited article in the disruption list was ranked as 12^th^ most disruptive. The title of the publication was “Histological grading and prognosis in breast cancer; a study of 1,409 cases of which 359 have been followed for 15 years” by Bloom and Richardson and was a very import paper in the formation of tumor grade as a prognostic factor for breast cancer. During the time of publication cancer grade was not widely discussed and the TMN staging system was the predominant source of prognostic information. This paper could be considered disruptive as it introduced new techniques to the prognostics of breast cancer care. 

The disruption list captured studies focusing on many different topics within breast cancer care. Important work in the development of breast cancer screening conducted by Shapiro et al. was represented. Further, prominent, early breast oncologists/pathologists such as CD Haagensen were included on the list with research vital to prognostics and surgical/radiologic treatment of breast cancer. Several early clinical trials in the use of hormonal therapy in breast cancer were also included amongst the most disruptive.

The most common study designs of disruptive publications were retrospective and prospective cohort studies but reviews, editorials, randomized control trials, case reports, basic science studies and others were represented in the top 100 list (supplementary Table 4). Of the 100 most disruptive studies captured in our results, eight were found to be unrelated to breast carcinoma care, a function of having general search terms. These unrelated studies had primary focuses of breast infection, papilloma, lymphoma, breast feeding, and milk fistula after biopsy.

**Figure 3:**
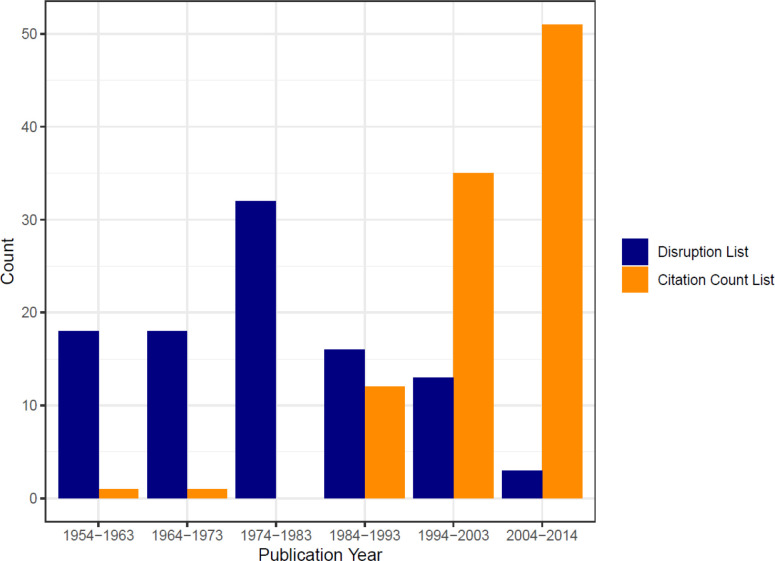
Frequency of Publication Year in the 100 Most Disruptive and Cited Papers

The correlation coefficients between disruption and citation count were low (R = 0.03). Scatter plot of citation count by disruption score is show in Figure 2. The distribution of studies identified in both disruption and citation count over time is shown in Figure 3. The median year of publication for the top 100 disruptive publications was 1977. The most common decade for most disruptive and most cited publications were between 1974-1983 and 2004-2013 respectively. 

## Discussion

In this paper we have searched 21 academic journals deemed important to the field of breast cancer care and identified the 100 most disruptive and 100 most cited publications. These disruptive studies were found in a variety of journals and had many different study designs. Disruptive studies skewed towards being older. The reason for this is uncertain but we suspect that it may be due to citation norms and democratization of information via the internet. This bias warrants further investigations and future evaluations using disruption scoring should take this into account. 

We demonstrate that disruption score correlates weakly with citation count and is therefore providing a unique metric to describe the academic impact of a publication. Increased dimensions in the way we characterize academic impact allows for a richer story to be told about the development of a field of scientific study. The disruption equation we utilized for this study captured a diverse array of study designs within the field of breast cancer care. Many of these papers where high impact and very clinically relevant while others were from small niches within breast cancer care. 

Take, for example, the publication with the highest disruption score, “The use of a specific hypofractionated radiation therapy regimen versus classical fractionation in the treatment of breast cancer: a randomized study of 230 patients.” In this study Baillet et al., (1990) compared the gold-standard of radiotherapy to a shorter duration, less fractionated regimen and found that the short course yielded similar long-term results. Studies such as the UK Standardization of Breast Radiotherapy Trials A and B, that developed and secured the technique of hypofractionation would be published nearly 20 years later (START Trialists’ Group et al., 2008). Despite being cited only 48 times in 30 years, the high disruption score of Baillet’s work reflects this publication’s early role in a shifting paradigm of breast radiotherapy. 

In contrast, Slamon’s et al., (2001) “Use of chemotherapy plus a monoclonal antibody against HER2 for metastatic breast cancer that overexpresses HE” is a highly cited publication with a low disruption score. This phase three clinical trial led to the popular adoption of trastuzumab in treatment regimens for Human Epidermal Growth Factor 2 (HER2) positive cancers. Though highly-cited, and undeniably impactful, it confirmed and cemented the role of monoclonal therapy in HER2 cancers. At the time of its publication HER2 was already identified as a potential drug target, and trastuzumab was demonstrated to be safe and effective. In juxtaposition to Baillet’s work on hypofractionated radiation, which initiated a divergence in radiotherapy dosing, Slamon et al added an important steppingstone on a linear pathway towards the use of trastuzumab. 

Impactfullness is difficult to measure and as previously stated the use of citation count as a surrogate for impact has come under heavy scrutiny. We do not claim in this study that disruption scoring is a replacement for citation count or a surrogate measure for impactfulness. Instead, we posit that the development of disruption scoring within the field of medicine can be used as an adjunctive measure in describing the bibliometric qualities of a paper and possibly give a richer description of the impact on any particular field.

It is clear that the current iteration of this disruption score captures papers that are truly disruptive to the field of breast cancer research but also those that have had little impact. Some studies that might be considered disruptive by experts in the field of breast cancer care are notably absents from the top 100 in this iteration. It is important to keep in mind that the large clinical trials that are often considered to be disruptive to the care of breast cancer patients are not typically disruptive to the field of study; they serve as extensions of work that has been previously conducted. Again, this is not to say that these works are not incredibly important and impactful but that these trials have characteristics of ending a line of inquiry rather than changing a line of inquiry.

Caution should be used when interpreting bibliometric studies, as ours, due to its static nature. This study demonstrates one “snapshot” in time but, as publications are increasingly cited their bibliometrics, change. This disruption score’s application within the field of health is relatively nascent and further refinement within the oncologic literature is warranted is warranted. 

In conclusion, disruption scoring is meant to capture research that changes thinking within a field of scientific inquiry. This is the first-time disruption scoring has been applied to breast cancer literature. Disruption offers a unique characteristic and we have shown that it is distinct from the predominant metric of citation count. Citation count is a useful bibliometric parameter and may serve to demonstrate a degree of impact but cannot fully encapsulate novelty or academic import. This idea has garnered more attention recently and many new bibliometric scores have been developed to compensate for the shortcomings of citation count. Disruption offers a unique characteristic and we have shown that it is distinct from the predominant metric of citation count. With further refinement, disruption in combination with other bibliometrics will be able to offer a more accurate and complete description of the impact of publications within the field of breast cancer care. Utilization in other oncologic specialties should be explored. 

## Author Contribution Statement

Miles Grunvald: Conceptualization, data collection/analysis interpretation, drafting of the article. Michael Williams: data collection/analysis interpretation, drafting of the article. Ruta Rao: Data curation. Cristina O’Donoghue data curation and editing. Adan Becerra: Data curation, methodology, project administration, review and editing. 

## Approval

This study was not conducted as part of any student thesis or project but was conducted under the Rush University Department of Surgery.
